# Molecular Diversity of Human Respiratory Syncytial Virus before and during the COVID-19 Pandemic in Two Neighboring Japanese Cities

**DOI:** 10.1128/spectrum.02606-22

**Published:** 2023-07-06

**Authors:** Takashi Ono, Koichi Hashimoto, Yohei Kume, Mina Chishiki, Hisao Okabe, Masatoki Sato, Sakurako Norito, Jumpei Aso, Mitsuru Sada, Izumi Mochizuki, Fumi Mashiyama, Naohisa Ishibashi, Shigeo Suzuki, Hiroko Sakuma, Reiko Suwa, Miyuki Kawase, Makoto Takeda, Kazuya Shirato, Hirokazu Kimura, Mitsuaki Hosoya

**Affiliations:** a Department of Pediatrics, Fukushima Medical University, Fukushima, Fukushima, Japan; b Department of Respiratory Medicine, Kyorin University School of Medicine, Tokyo, Japan; c Department of Pediatrics, Ohara General Hospital, Fukushima, Fukushima, Japan; d Department of Pediatrics, Hoshi General Hospital, Koriyama, Fukushima, Japan; e Department of Virology 3, National Institute of Infectious Diseases, Tokyo, Japan; f Department of Microbiology, Graduate School of Medicine and Faculty of Medicine, The University of Tokyo, Tokyo, Japan; g Gunma Paz University, Graduate School of Health Sciences, Takasaki, Gunma, Japan; Pontificia Universidad Católica de Chile

**Keywords:** evolution, molecular epidemiology, respiratory syncytial virus

## Abstract

Human respiratory syncytial viruses (HRSVs) are divided into subgroups A and B, which are further divided based on the nucleotide sequence of the second hypervariable region (HVR) of the attachment glycoprotein (G) gene. Understanding the molecular diversity of HRSV before and during the coronavirus disease 2019 (COVID-19) pandemic can provide insights into the effects of the pandemic on HRSV dissemination and guide vaccine development. Here, we analyzed HRSVs isolated in Fukushima Prefecture from September 2017 to December 2021. Specimens from pediatric patients were collected at two medical institutions in neighboring cities. A phylogenetic tree based on the second HVR nucleotide sequences was constructed using the Bayesian Markov chain Monte Carlo method. HRSV-A (ON1 genotype) and HRSV-B (BA9 genotype) were detected in 183 and 108 specimens, respectively. There were differences in the number of HRSV strains within clusters prevalent at the same time between the two hospitals. The genetic characteristics of HRSVs in 2021 after the COVID-19 outbreak were similar to those in 2019. HRSVs within a cluster may circulate within a region for several years, causing an epidemic cycle. Our findings add to the existing knowledge of the molecular epidemiology of HRSV in Japan.

**IMPORTANCE** Understanding the molecular diversity of human respiratory syncytial viruses during pandemics caused by different viruses can provide insights that can guide public health decisions and vaccine development.

## INTRODUCTION

Human respiratory syncytial viruses (HRSVs) are a major cause of lower respiratory tract infections, particularly in children and elderly individuals (1). A meta-analysis estimated that approximately 33.1 million episodes of acute lower respiratory tract infections were caused by HRSVs worldwide in 2015, with 3.2 million cases requiring hospitalization and approximately 60,000 deaths in children under the age of 5 years (2). In the United States, approximately 14,000 HRSV infection-related deaths of individuals aged 65 years and older are reported annually (3, 4). Palivizumab, a monoclonal antibody against the HRSV fusion (F) protein, is the sole drug used to treat HRSV infection and is indicated only for high-risk pediatric patients to alleviate severe HRSV infections (5). Owing to the high disease burden, along with a lack of therapeutic agents and universal vaccines, HRSV infection is considered a global health problem (6).

Human respiratory syncytial viruses belong to the family *Pneumoviridae*, which consists of nonsegmented negative-sense single-stranded enveloped RNA viruses. They are divided into major subgroups A and B based on monoclonal antibody studies (7). Both subgroups exhibit 1- to 3-year cyclic variations worldwide (8, 9). An epidemiological study has shown that both subgroups can co-occur during a single epidemic period; however, even in the event of coendemicity, temporal and geographic aggregations can occur (10). Antigenic differences between the HRSV subgroups possibly contribute to their reinfection ability (11).

Among all relevant HRSV genes, the attachment glycoprotein gene (*G*) has the highest number of variations that can affect viral infection and transmission (12). Currently, HRSV typing is based on the nucleotide sequence of the second hypervariable region (HVR) of *G* (13). Phylogenetic analysis based on the alignment of HRSV *G* sequences, including those from HRSV-A and HRSV-B reference strains, has been conducted (9). HRSV-A comprises 15 genotypes (GA1 to -7, SAA1, NA1 to -4, CB-A, ON1, and ON2) (9, 13–18), and HRSV-B comprises 29 genotypes (GB1 to -5, SAB1 to -4, URU1, URU2, CB-B, BA-C, CB-1, THB, and BA1 to -14) (9, 13, 14, 16, 19–27). An eight-nation study showed that BA9 HRSV-B and ON1 HRSV-A were the predominant cocirculating genotypes in most regions during 2017 and 2018 (28). According to a nationwide Japanese survey during 2012 to 2015, ON1 replaced NA1 as the predominant HRSV-A genotype during 2014 and 2015. BA9 and BA10 were the predominant HRSV-B genotypes during 2013 and 2014, whereas only BA9 was detected during 2014 and 2015 (29).

Although similar genotypes of HRSVs are prevalent globally, further analysis of the genotypes at the lineage and cluster levels could provide deeper insights into the molecular diversity of HRSVs in different regions. Several phylogenetic studies from various countries have been published, but only a few have made in-depth comparisons of the molecular epidemiology of HRSVs in small communities.

Similar to other countries, Japan had fewer HRSV infection outbreaks in 2020 than in previous years, perhaps because of a reduction in human movement and the implementation of infection prevention and control measures in December 2019 as coronavirus disease 2019 (COVID-19), which originated in Wuhan, China, progressed to become a global pandemic (30, 31). Therefore, it is reasonable to investigate the evolution of HRSVs before and after their re-emergence in 2021.

We performed an evolutionary/epidemiological study based on the second HVR of *G* to investigate the temporal and geographic changes in circulating HRSVs at two medical institutes in neighboring cities in Fukushima Prefecture, Japan, from September 2017 to December 2021.

## RESULTS

### Study area.

This study involved two medical institutions in Fukushima Prefecture (referred to here as hospital O, in Fukushima City, and hospital H, in Koriyama City). The distance between the two institutions is approximately 40 km. The two cities are connected by highways and railways; therefore, a large number of people regularly visit these cities. However, the cities belong to different medical regions; each city has different institutions where comprehensive medical care, ranging from disease prevention to inpatient treatment, is provided. Each city has a population of approximately 300,000 individuals, including a pediatric population of 30,000 to 40,000 individuals. At each institution, approximately 350 patients with respiratory tract infections are hospitalized per year.

### Detection and epidemiological characterization of HRSV.

**FIG 1:**
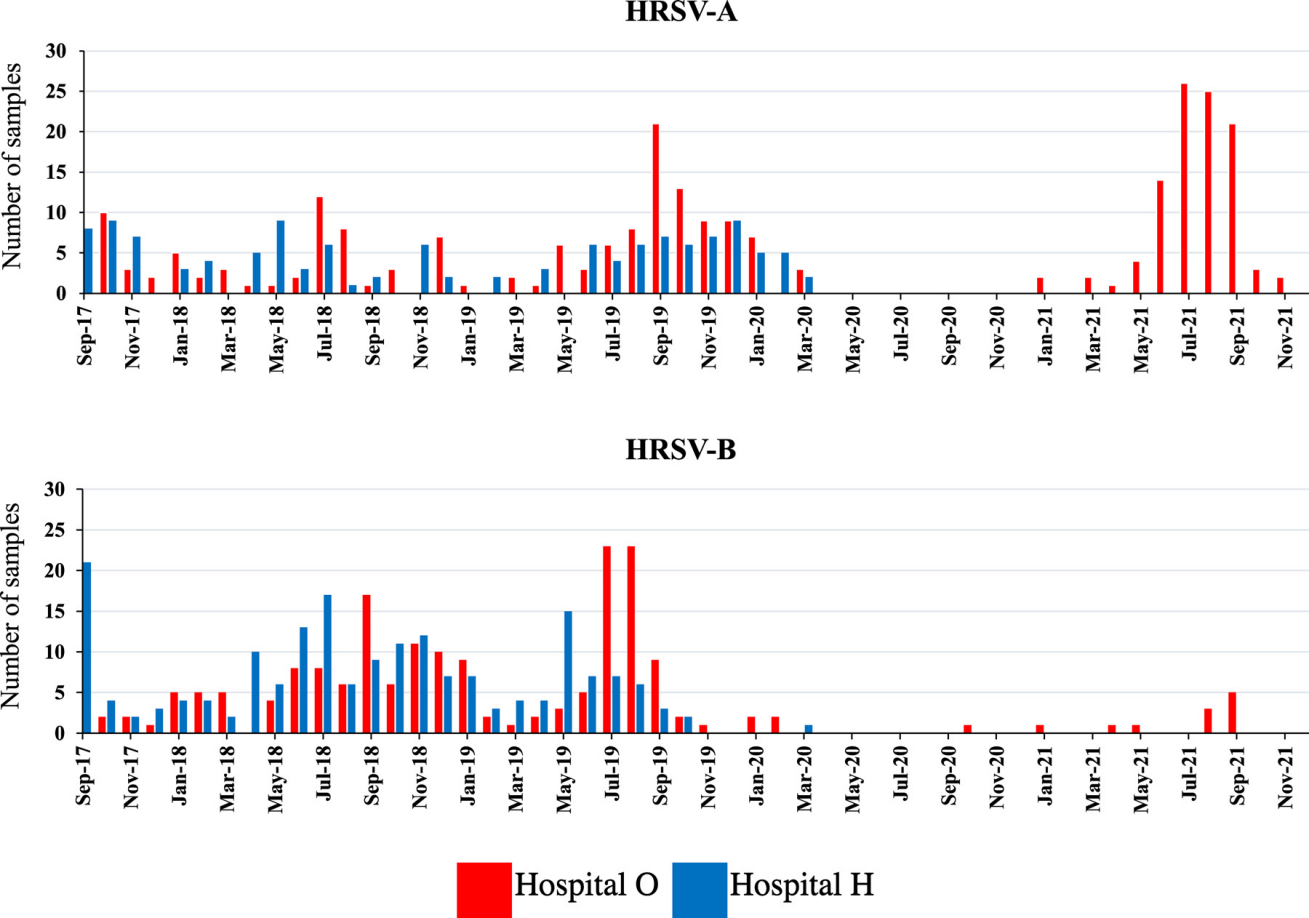
Detection of HRSV-A and HRSV-B in the hospitals included in the study. From September 2017 to March 2020, 667 specimens tested positive for HRSV in both hospitals, and from April 2020 to December 2021, 112 specimens tested positive for HRSV in hospital O.

From September 2017 to March 2020, of the 1,838 patients admitted to the two hospitals, specimens collected from 667 patients tested positive for HRSV using quantitative PCR (qPCR). Of these HRSV-positive specimens, five were collected at the beginning of each month at each hospital; when fewer than five specimens were collected per month, all specimens were included. Thus, 219 specimens were included in this study. The second HVR of *G* was successfully sequenced in 203 specimens. From April 2020 to December 2021, specimens collected only at hospital O were included. Of the 492 patients admitted to hospital O, 112 tested positive for HRSV using qPCR. The second HVR of *G* was successfully sequenced in 87 specimens. Therefore, 290 specimens were included in the final analysis. Of the 290 specimens, 183 were HRSV-A positive and 108 were HRSV-B positive. One specimen was from a patient infected with both HRSV-A and HRSV-B. The number of HRSV-A- and HRSV-B-positive specimens detected using qPCR is shown in Fig. 1.

Next, we analyzed patient demographic and clinical characteristics based on HRSV subgroups in the 203 specimens collected at hospitals O and H from September 2017 to March 2020 and successfully sequenced. One specimen from a patient infected with both HRSV-A and HRSV-B was excluded; therefore, the comparison between HRSV-A and HRSV-B involved 202 patients (Table 1). Although the age groups differed significantly between the subgroups, no significant differences were found in sex, choice of hospital, or clinical symptoms.

**TABLE 1:** Patient demographic and clinical characteristics based on HRSV subtypes detected at hospitals O and H during 2017 to 2020 (*n* = 202)^*a*^

Characteristic	Total (*n* = 202)		HRSV-A (*n* = 99)		HRSV-B (*n* = 103)		*P*
	No.	%	No.	%	No.	%	
Age (yrs)							0.040
<1	97	48.0	50	50.5	47	45.6	
1–5	100	49.5	44	44.4	56	54.4	
>5	5	2.5	5	5.1	0	0.0	
Sex							0.685
Male	107	53.0	51	51.5	56	54.4	
Female	95	47.0	48	48.5	47	45.6	
Hospital							0.789
Hospital O	106	52.5	51	51.5	55	53.4	
Hospital H	96	47.5	48	48.5	48	46.6	
Clinical characteristic							
Fever	168	83.2	79	79.8	89	86.4	0.209
Cough	195	96.5	95	96.0	100	97.1	0.661
Rhinorrhea	160	79.2	80	80.1	80	77.7	0.583
Wheeze	56	27.7	27	27.3	29	28.2	0.889

a This analysis included 202 specimens collected at two hospitals from September 2017 to March 2020 and sequenced. One sample from a patient coinfected with HRSV-A and HRSV-B was excluded. Subgroups were compared using the chi-square test or Fisher’s exact test. Statistical significance was set at a *P* value of <0.05.

We analyzed patient demographic and clinical characteristics by sample collection year with 135 specimens collected from September 2017 to December 2021 in hospital O (Table 2). HRSV-B was excluded from the analysis because of the small number of specimens. A comparison of the specimens collected from 2017 to 2020 and in 2021 revealed no differences in patient age, sex, or clinical symptoms.

**TABLE 2:** Demographic and clinical characteristics of patients infected with HRSV-A in hospital O (*n* = 135)^*a*^

Characteristic	Total (*n* = 135)		2017–2020 seasons (*n* = 52)		2021 season (*n* = 83)		*P*
	No.	%	No.	%	No.	%	
Age (yrs)							0.891
<1	4	3.0	2	3.9	2	2.4	
1–5	68	50.4	26	50.0	42	50.6	
>5	63	46.7	24	46.2	39	47.0	
Sex							0.504
Male	73	54.1	30	57.7	43	51.8	
Female	62	45.9	22	42.3	40	48.2	
Clinical characteristic							
Fever	105	77.8	42	80.8	63	75.9	0.508
Cough	131	97.0	51	98.1	80	96.4	0.573
Rhinorrhea	115	85.2	44	84.6	71	85.5	0.883
Wheeze	40	29.6	12	23.1	28	33.7	0.187

a This analysis included 135 specimens collected at hospital O from September 2017 to December 2021 and sequenced. The collection periods were compared using the chi-square test or Fisher’s exact test. Statistical significance was set at a *P* value of <0.05.

### Subgroups and genotypes.

Genotypes of all HRSV-A and HRSV-B clinical strains were determined by identifying closely related strains of each clinical strain using the Basic Local Alignment Search Tool (BLAST) (32). The genotypes were confirmed through the findings of nucleotide sequencing and phylogenetic tree analysis as described below. HRSV-A clinical strains were classified as ON strains because of the presence of a 72-base repeat sequence in the second HVR of *G*. In addition, based on the results of phylogenetic tree analysis using the maximum-likelihood (ML) method, HRSV-A clinical strains were clustered with ON1 strains identified in the BLAST analysis (see Fig. S1 in the supplemental material). In the Bayesian phylogenetic tree, the clinical strains branched off from ON1 strains (Fig. S2). HRSV-B clinical strains were classified as BA strains because of the presence of a 60-base repeat sequence in the second HVR of *G*. Furthermore, based on the phylogenetic tree analysis using the ML method, HRSV-B clinical strains were clustered with BA9 strains identified in the BLAST analysis (Fig. S3). In the Bayesian phylogenetic tree, the clinical strains branched off from BA9 strains (Fig. S4).

### HRSV-A phylogeny.

**FIG 2:**
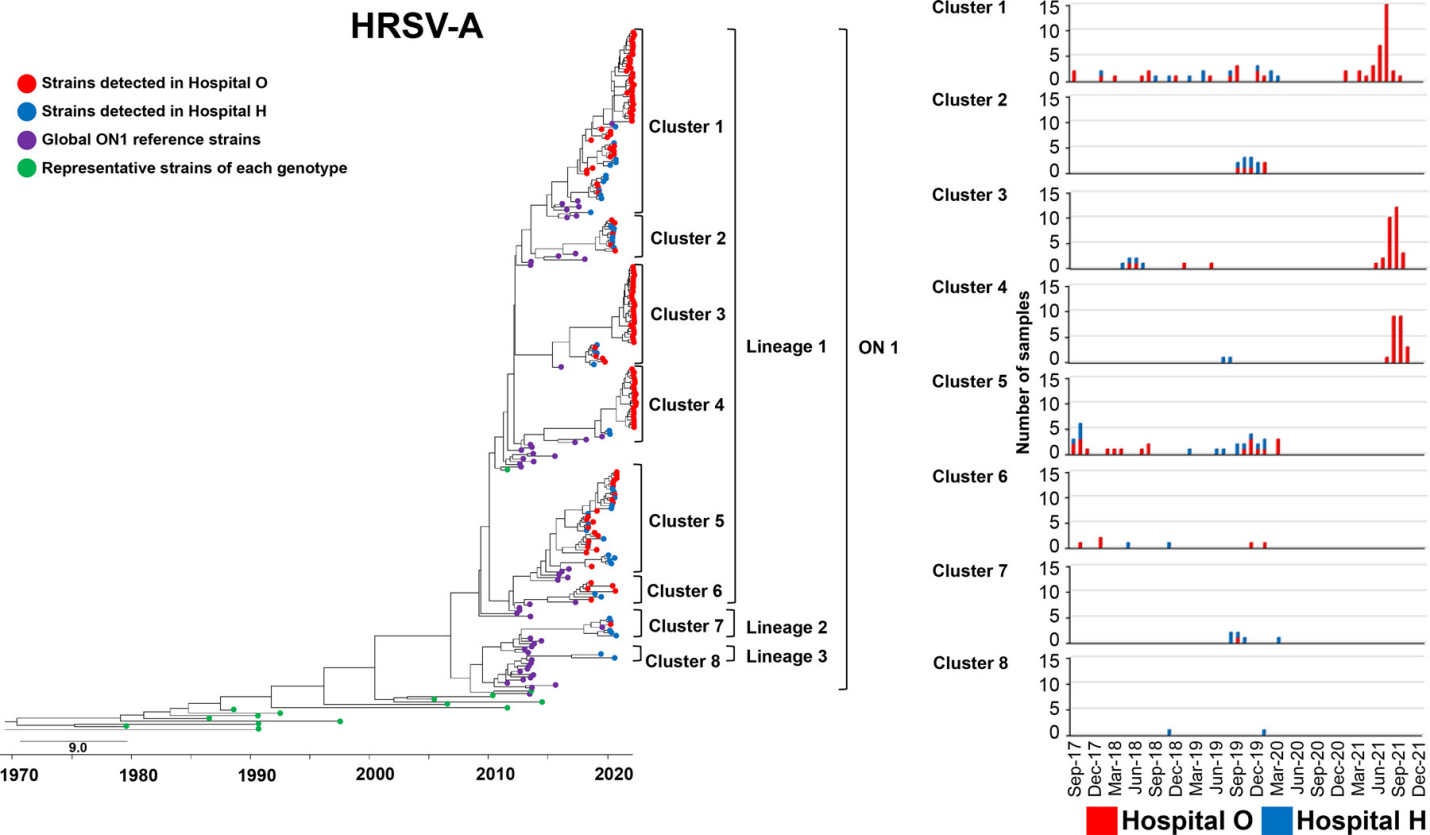
Time-scaled maximum clade credibility tree using the best model (general time-reversible [GTR] model) of the second HVR of *G* from HRSV-A constructed using the Bayesian MCMC method under the exponential molecular clock. A 10-year timeline is shown below the tree. Strains detected at hospital O are shown in red, and those detected at hospital H are shown in blue. Strains circulating in different countries are shown in purple. Representative strains of each genotype are shown in green. On the right are the number and detection time of HRSVs detected in each cluster at hospitals O and H.

We constructed a phylogenetic tree of our HRSV-A clinical strains, representative strains of each genotype, strains closely related to our clinical strains, and ON1 strains detected worldwide (Fig. 2). The evolutionary rate of HRSV-A strains included in this analysis is shown in Table S1. Considering the four recognized ON1 lineages (15, 33), most clinical strains belong to lineage 1, and so do most of the ON1 strains circulating worldwide. Six clusters were present in lineage 1. Additionally, a few strains were clustered in lineage 2 or 3 (one cluster each). Eight clusters (numbered 1 to 8) were identified. Clusters 1, 5, and 6 were commonly detected during 2017 to 2019. Clusters 2 and 7 were detected only in 2019. All HRSVs detected at hospital O in 2021 were in clusters 1, 3, and 4, forming genetically close populations within each cluster (Table S2). The pairwise distance per cluster of HRSV-A detected at hospitals O and H during 2017 to 2021 is shown in Table S3. Details of the HRSV-A strains are shown in Fig. S2.

### HRSV-B phylogeny.

**FIG 3:**
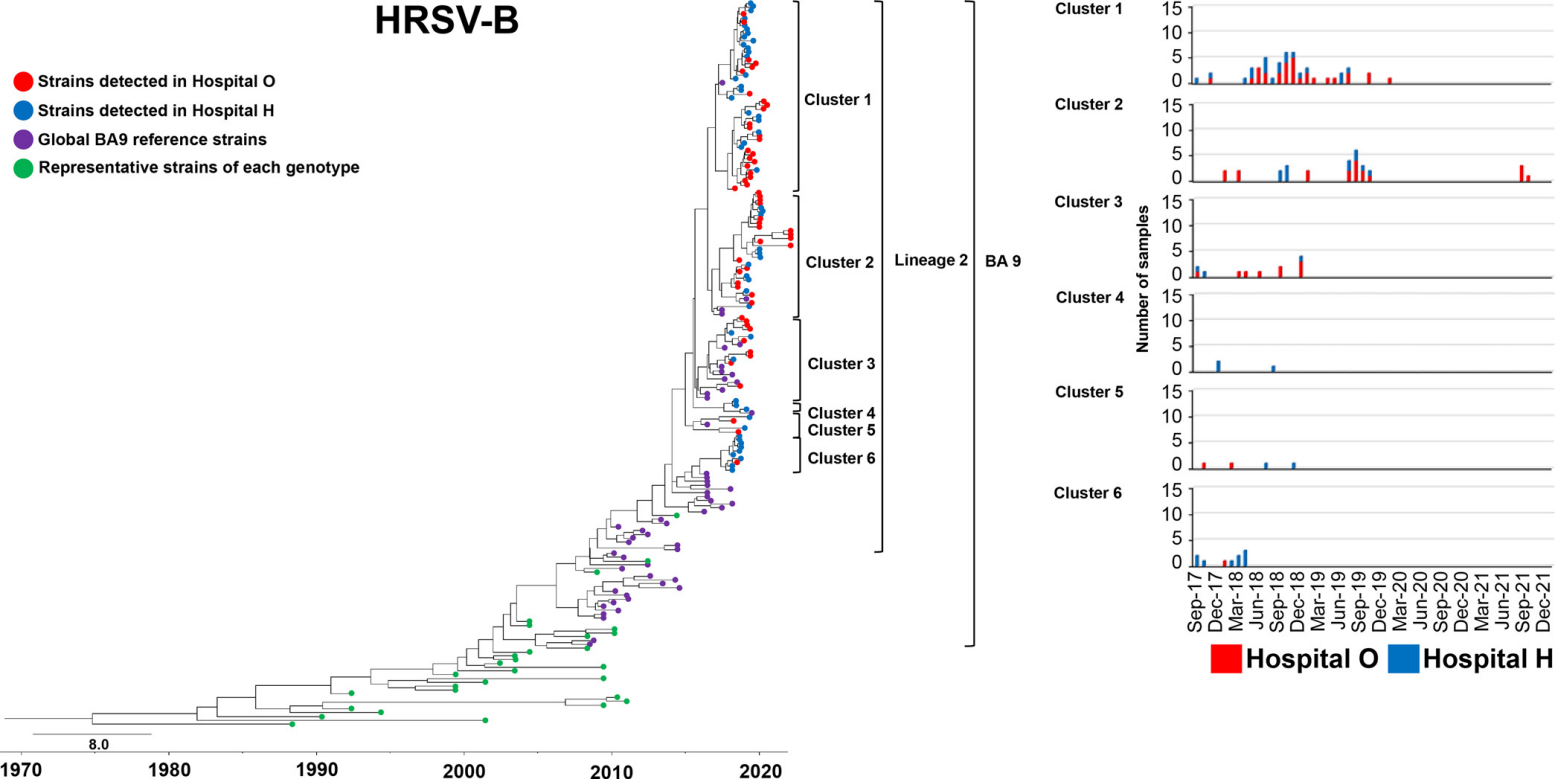
Time-scaled maximum clade credibility tree using the best model (GTR model) of the second HVR of *G* from HRSV-B constructed using the Bayesian MCMC method under the exponential molecular clock. A 10-year timeline is shown below the tree. Strains detected at hospital O are shown in red, and those detected at hospital H are shown in blue. Strains circulating in different countries are shown in purple. Representative strains of each genotype are shown in green. On the right are the number and detection time of HRSVs detected in each cluster at hospitals O and H.

We constructed a phylogenetic tree for HRSV-B strains using the same approach (and the relevant control strains) (Fig. 3). The evolutionary rate of HRSV-B strains included in this analysis is shown in Table S1. BA9 is currently divided into three lineages (34); however, all our clinical strains belonged to lineage 2, similar to most BA9 strains detected worldwide recently. Six clusters belonged to lineage 2. Clusters 1 and 2 were commonly detected during 2017 to 2019, whereas clusters 3, 4, and 5 were detected only in 2017 and 2018. Cluster 6 was detected only in 2017. In 2021, all four HRSVs detected at hospital O were in cluster 2, forming genetically close populations within each cluster (Table S2). The pairwise distance per cluster of HRSV-B detected at hospitals O and H during 2017 to 2021 is shown in Table S3. Details of the HRSV-B strains are presented in Fig. S4.

### Genome networks.

**FIG 4:**
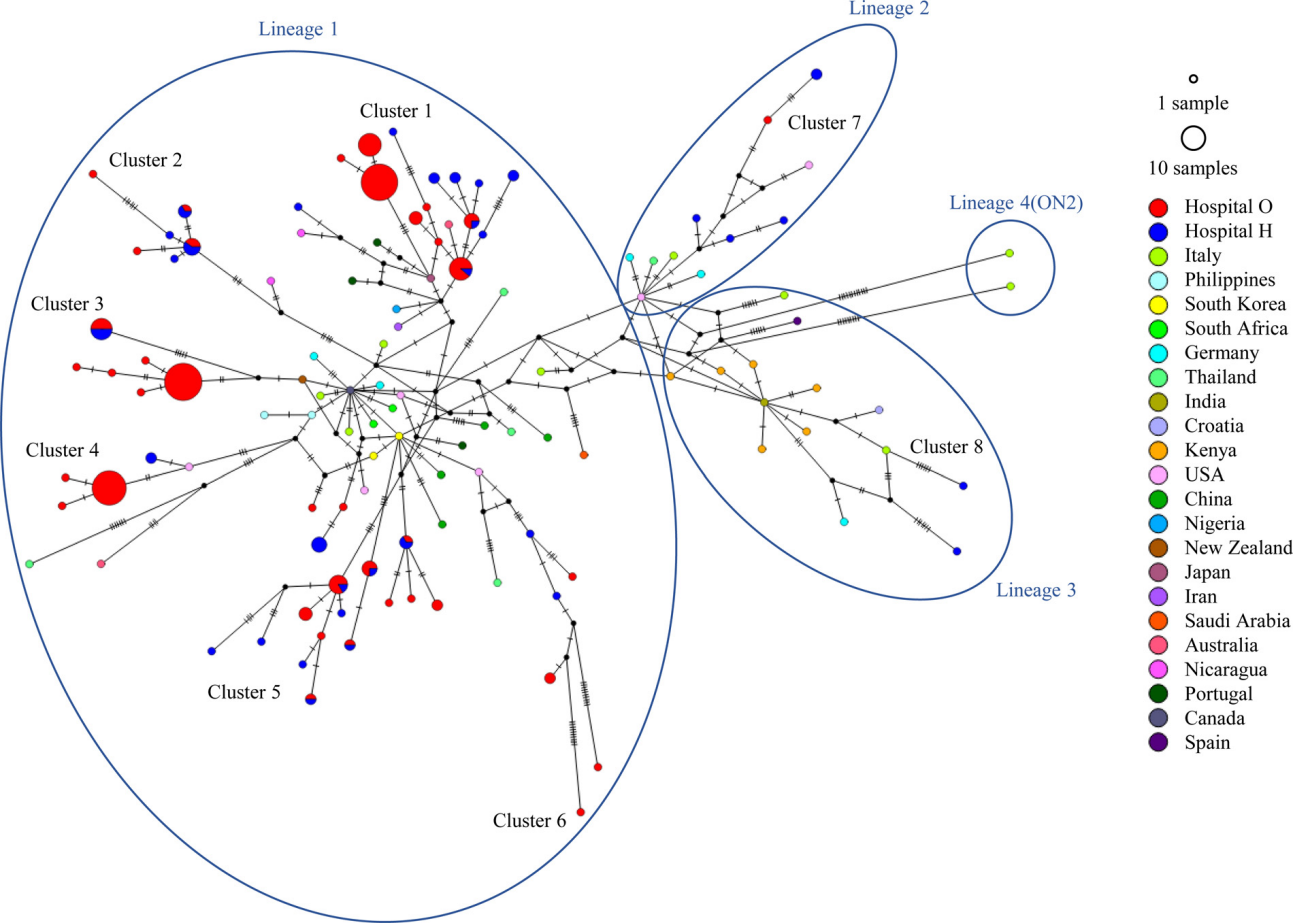
HRSV-A ON1 median-joining network based on the sequences of the second HVR of *G* depicting the relationships between clusters and lineages. The internodal line lengths are proportional to the number of mutations. The size of the circles represents the number of strains with matching sequences. Strains circulating in different countries are color coded. Strains detected at hospital O are shown in red, and those detected at hospital H are shown in blue.

**FIG 5:**
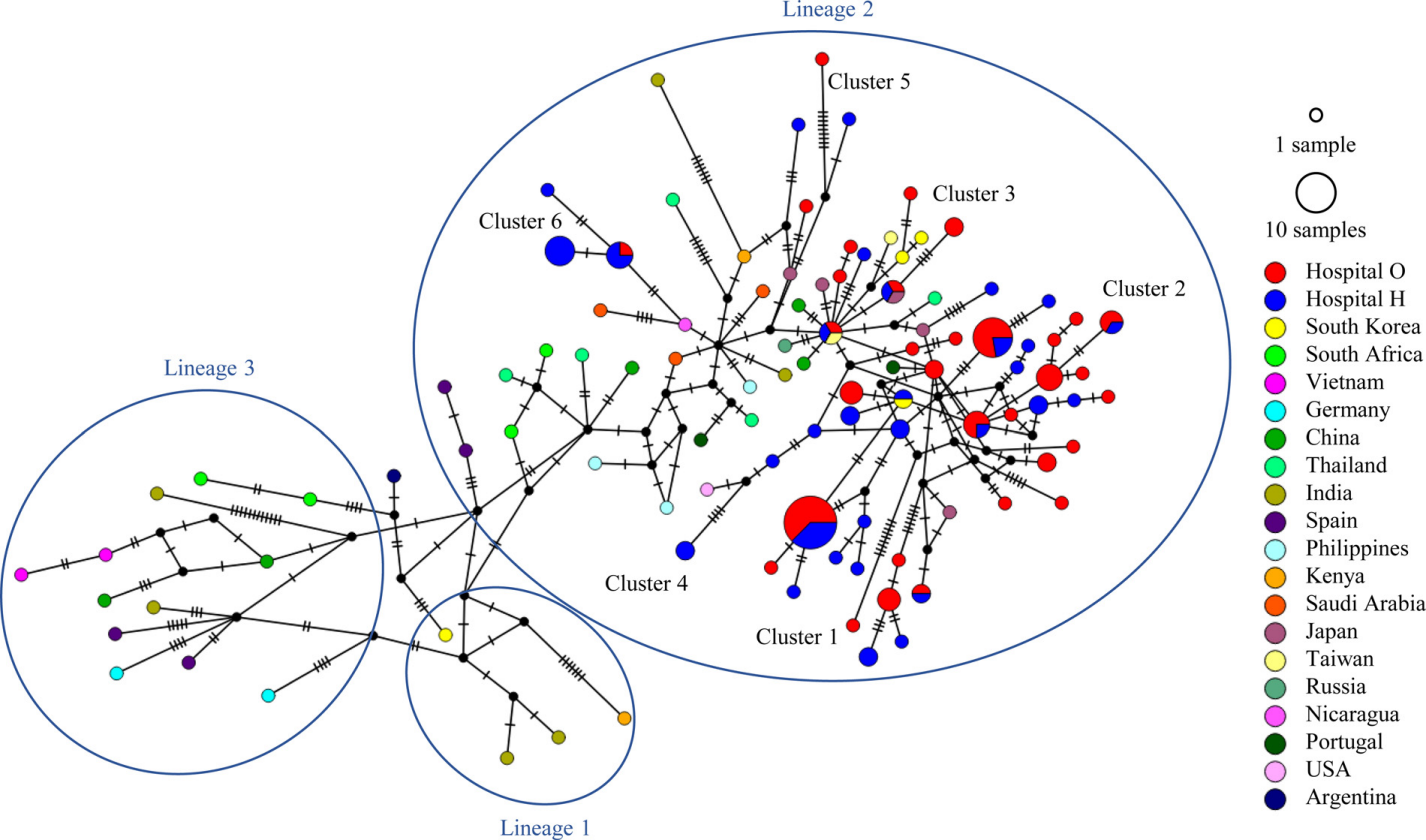
HRSV-B BA9 median-joining network based on the sequences of the second HVR of *G* depicting the relationship between clusters and lineages. The internodal line lengths are proportional to the number of mutations. The size of the circles represents the number of strains with matching sequences. Strains circulating in different countries are color coded. Strains detected at hospital O are shown in red, and those detected at hospital H are shown in blue.

We included clinical strains of HRSV-A and HRSV-B, strains closely related to our clinical strains, and ON1 and BA9 strains detected worldwide in the data set and performed a network analysis on each of them (Fig. 4 and 5). For HRSV-A, as shown in the aforementioned phylogenetic tree, most clinical strains were in lineage 1, and eight clusters were easily distinguished. Five clinical strains, including 1 from hospital O and 4 from hospital H, belonged to lineage 2, whereas two clinical strains from hospital H belonged to lineage 3. Most ON1 strains detected in other countries also belong to lineage 1. Regarding HRSV-B, all clinical strains were from lineage 2, similar to most BA9 strains detected in other countries.

## DISCUSSION

In this study, we investigated the molecular diversity and epidemiology of HRSVs collected at two hospitals in neighboring cities of Fukushima Prefecture using the second HVR of *G*. We compared the prevalent clusters between the cities to determine whether there are differences in the molecular diversity of HRSVs even between neighboring regions. We also assessed the evolution of HRSV before and during the COVID-19 pandemic. We found that the directionality of both HRSV-A and HRSV-B clinical strains was similar to that of global epidemic strains. The clinical strains formed clusters with unique characteristics, as indicated by our extensive phylogenetic and network analyses. We observed differences in the epidemic status and epidemic clusters of both HRSV-A and HRSV-B clinical strains even between neighboring regions. The re-emergence of HRSV in 2021 after the COVID-19 outbreak possibly originated from endogenous circulating HRSVs.

In 2017 and 2018, the globally predominant HRSV genotypes were ON1 for HRSV-A and BA9 for HRSV-B, with the latter being predominant in most countries (28, 35–41). The HRSV genotypes BA and ON1 showed 60- and 72-bp overlaps, respectively, in the second HVR of *G* because of gene duplication events (22, 40). These HRSVs with overlapping genotypes spread worldwide within a few years of identification, likely because the global population is immunologically naive (42). The emergence of a greater number of *G* variants can be attributed to the selective pressure exerted by host immunity, as a way to escape cross-protective immune responses (43, 44). This hypothesis is supported by the results of a longitudinal whole-genome sequencing study of HRSV specimens collected from infants with severe combined immunodeficiency before and after bone marrow transplantation, which showed increased *G* diversity after immune reconstitution (45).

All HRSV-A and HRSV-B clinical strains in this study were identified as ON1 and BA9, respectively. Most HRSV-A clinical strains were in lineage 1, whereas a few strains were in lineage 2 or 3; in contrast, all HRSV-B clinical strains were in lineage 2. Strains of the same genotypes circulating in other countries were predominantly in lineage 1 for ON1 and lineage 2 for BA9. Our results suggest that the epidemic pattern in a small community shows a trend similar to that observed worldwide. Based on our Markov chain Monte Carlo (MCMC) phylogenetic analysis, a graph was plotted for each cluster with the number of clinical strains and time of detection at hospitals O and H. Together, our results showed differences in the detection time and duration of the clusters of both HRSV-A and HRSV-B prevalent at hospitals O and H.

Peret et al. (46) analyzed HRSV community circulation patterns and reported that several strains are likely to be introduced into each community each year and cocirculate with endemic strains, with local factors determining the predominant strains. A previous study reported that both community factors and global spread can affect the circulation of strains in a community (13). In a study of 15 outbreaks of infection with HRSV-B BA in Kilifi, Kenya, the viruses circulating in a year were not from a single phylogenetic cluster but from multiple clusters. In contrast, sequences of viruses from successive epidemics were more clustered, suggesting the persistence of some variants between HRSV epidemic seasons (47). Annual HRSV infection outbreaks are caused by variants resulting from locally evolved clades, not by distantly introduced viruses (48, 49). However, the possibility that closely related viruses may be introduced from other locations each year cannot be ruled out (50). In the present study, we identified clusters that disappeared after one or two seasons and clusters that were persistently detected. Therefore, HRSVs that mutate in each region and are introduced from other regions could lead to an epidemic that may be repeated with the emergence of a mutant virus adapted to the environment of a particular region and its spread globally. In Japan, HRSVs are prevalent in the fall and winter; however, during 2017 to 2019, they were prevalent in the summer and fall (51). Our results also revealed an HRSV epidemic during this period. We found clusters that were detected every year as well as some that were detected in only 1 year. Each year, multiple strains are introduced from other regions or circulate endogenously within a region. In neighboring cities, regional factors such as the immune status of the population affect the circulation of HRSVs and produce clusters with distinctive characteristics. In 2021, HRSVs from hospital O were closely related to the cluster detected in 2019, with many strains of the same sequence type or with a low pairwise distance (Table S2). This finding suggests that the HRSV strains that circulated in 2021 after the end of the HRSV epidemic in 2019 and following a nonendemic period in 2020 continued to circulate within the local community and re-emerged.

According to a national HRSV surveillance in Japan (RSV infection cases reported per sentinel weekly by the National Institute of Infectious Diseases, Japan [https://www.niid.go.jp/niid/ja/10/2096-weeklygraph/1661-21rsv.html]), there was no HRSV epidemic in 2020, when nonpharmaceutical interventions (NPIs) were used to prevent human movement and control the COVID-19 pandemic; in contrast, a major HRSV epidemic, whose magnitude was greater than that observed in previous years, occurred in 2021. In South Africa, France, Israel, Australia, and the United States, COVID-19-related NPIs markedly reduced RSV activity; however, increased RSV activity was observed when the NPIs were lifted (52–57).

As Japan continued to restrict international travel after 2020 as a measure to prevent the spread of infection, it is unlikely that the HRSV strain that caused the outbreak in 2021 was introduced from another country. Our phylogenetic tree analysis confirmed the evolution from clusters endemic to each region in the previous year, especially for HRSV-A, suggesting that strains circulating within these areas re-emerged. The incidence of HRSV infection in children aged 2 years and above increased in 2021 compared with that in previous years in Japan (58). This may be because of the lack of exposure to HRSVs in 2020, leading to the formation of an immunologically naive population and an increased number of susceptible infants, which may have caused the epidemics of HRSVs, with a few genetic mutations. In a study on HRSV epidemic in Australia after the ease of COVID-19-related restrictions, whole-genome analysis showed that the genetic diversity of HRSVs decreased significantly after the start of the COVID-19 pandemic, suggesting that although the origin and detection time of the epidemic strains were not clear, they may have been circulating within the country (59). A study examining the evolutionary dynamics of seasonal influenza virus circulating worldwide during the COVID-19 pandemic revealed that genetic diversity had declined and that most residual transmission lineages were independently maintained and circulated within small regions (60). The results of these studies support our observations.

This study had some limitations. First, the number of specimens analyzed was limited, and some specimens could not be sequenced because of small sample volume. Second, as we observed differences in the number of HRSV strains in clusters prevalent at the same time in neighboring cities, we consider that HRSVs in each region may be diverse. However, this could not be proven in the present study because the number of specimens was small and specimens from only two regions were analyzed. To prove this hypothesis, we need to increase the number of specimens and include samples from other regions in the same prefecture as well as other prefectures in Japan for comparison. Third, the number of global strains included in the analysis was limited. It should be considered that our results may differ from those of other studies that included different strains. However, this is unlikely, because the reference strains used in this study represent all current major genotypes. Fourth, the second HVR of *G* was analyzed using a method adopted in most epidemiological studies. However, this approach has not been used in all studies; some studies have used the ectodomain of *G* for improved HRSV genotype classification (61, 62), whereas others have used whole-genome sequencing (63). However, *G* remains the basis for understanding the molecular epidemiology of HRSVs, and analysis of the same gene region allows comparison of results among studies.

In conclusion, by analyzing the second HVR of *G*, we found that the molecular epidemiology of HRSVs in neighboring cities in Japan is similar to that in other parts of the world. We found differences in the number of HRSV strains within clusters that were simultaneously prevalent at the same time in each region. Given that the genetic characteristics of HRSVs circulating in 2021 after the COVID-19 outbreak were similar to those of strains circulating in 2019, HRSVs in the same cluster could circulate for several years, thereby initiating an epidemic cycle, in addition to the introduction of strains from other areas. These results substantially add to our knowledge of the molecular epidemiology of HRSV infection. Continued surveillance is required to gain deeper insights into the molecular diversity of HRSVs.

## MATERIALS AND METHODS

### Patients and specimens.

Nasopharyngeal swab specimens were collected from 2,330 patients admitted to the pediatric wards of the two hospitals (1,500 in hospital O and 830 in hospital H) for respiratory tract infections (upper respiratory tract infections, lower respiratory tract infections, croup syndrome, and otitis media) between September 2017 and December 2021. Specimens from patients from whom informed consent could not be obtained were excluded from the study. The specimens were immediately stored in a freezer at −80°C in the Fukushima Medical University laboratory until analysis.

### RNA extraction and real-time quantitative reverse transcription-PCR.

Viral RNA was extracted from 140 μL of each specimen using the QIAamp viral RNA minikit (Qiagen, Valencia, CA, USA) according to the manufacturer’s instructions. Thereafter, reverse transcription-qPCR (RT-qPCR) was performed using the AgPath-ID one-step RT-PCR reagent (Thermo Fisher Scientific, Waltham, MA, USA) as described previously (64).

### Sequencing.

Viral cDNA was generated from the extracted RNA using the PrimeScript RT-PCR kit (TaKaRa Bio, Otsu, Japan). The second HVR of *G* was amplified using the PrimeScript RT-PCR kit (TaKaRa Bio) as described previously (13, 65). The primers used in the experiment were as follows: forward primer F1 (5′-CAACTCCATTGTTATTTGCC-3′) with reverse primers nRSAG (5′-TATGCAGCAACAATCCAACC-3′) and nRSBG (5′-GTGGCAACAATCAACTCTGC-3′). The PCR conditions were as follows: 95°C for 2 min; 40 cycles of 94°C for 1 min, 54°C for 1 min, and 72°C for 2 min; and a final extension step at 72°C for 7 min. After electrophoresis, the PCR products were purified using the MinElute PCR purification kit and MinElute gel extraction kit (both from Qiagen). Next, they were labeled using the BigDye Terminator V3.1 cycle sequencing kit (Thermo Fisher Scientific) and sequenced using the ABI 3730xl DNA analyzer (Applied Biosystems, Foster City, CA, USA).

### Genotyping.

The nucleotide sequence length of HRSV-A clinical strains was 336 bp, whereas that of HRSV-B strains was 324 bp. BLAST analysis was performed on this segment to find closely related strains of all clinical strains and to identify genotypes.

### RSV sequence database.

To understand the molecular evolution of the RSV G protein, we downloaded the nucleotide sequence of the second HVR of HRSV *G* from GenBank. We used the 1956 strain Long (JX198112) and 1961 strain A2 (KT992094) of HRSV-A and the 1962 strain CH18537 (JX198143) of HRSV-B as outgroups. Other strains incorporated into the data set were as follows: 15 HRSV-A strains and 29 HRSV-B strains were selected as representatives of each genotype, 16 HRSV-A strains and 6 HRSV-B strains were selected as close relatives from the BLAST research results, and 37 HRSV-A strains and 48 HRSV-B strains were selected as global strains based on previous studies (9, 14–27, 34–39, 46, 66–72). The data are presented in Table S4.

### Classification of lineages and clusters.

For lineage classification, the strains classified in each lineage were incorporated into the data set and classified based on their positions in the Bayesian phylogenetic tree as described by Hirano et al. (15) for HRSV-A and Haider et al. (34) for HRSV-B.

Furthermore, cluster classification of each clinical strain was performed using a BLAST search to identify closely related strains, which were then incorporated into the data set and classified based on their position in the Bayesian phylogenetic tree.

### Phylogenetic analyses.

Nucleotide sequences of the second HVR of *G* were aligned and edited using ClustalW in MEGA version 7.0.26 (73). jModelTest version 2.1.10 (74) was used to select the appropriate nucleotide substitution model. First, a phylogenetic tree was constructed using the ML method with 1,000 bootstrap replicates using MEGA version 7.0.26. Thereafter, PA phylogenetic analysis was performed using the Bayesian MCMC method in BEAST version 2.4.8 (75). We compared four clock models (strict clock, relaxed clock exponential, relaxed clock log-normal, and random local clock) with two tree prior models (coalescent constant population and coalescent exponential population) to identify the best model (76). To verify the convergence of the MCMC method, we used the path-sampling method using the path sampler included in BEAST. To confirm the convergence of the MCMC method, we further evaluated the effective sample size using Tracer version 1.7.1 (77) and confirmed that the value of all parameters exceeded 200. The first 10% of the phylogenetic data obtained were excluded (burn-in) because of potential unreliability, and the best phylogenetic tree was designed using TreeAnnotator in BEAST. MCMC phylogenetic trees were constructed using FigTree version 1.4.0 (http://tree.bio.ed.ac.uk/software/figtree/).

### Median-joining network analysis.

To evaluate the geographical determinants of HRSV evolution, data sets were converted to NEXUS files using DnaSP version 6.12.03 (78), and information regarding the area was added to Word 2019 version 1808 and analyzed using PopART (full-feature software for haplotype network construction) version 1.7 (79), with the median-joining algorithm.

### Statistical analysis.

Demographic and clinical characteristics based on the HRSV subgroups and collection periods were compared using the chi-square test or Fisher’s exact test. Statistical significance was set at a *P* value of <0.05. All analyses were performed using IBM SPSS Statistics version 27 (SPSS Inc., Chicago, IL, USA).

### Ethics statement.

This study was approved by the Ethics Committee of Fukushima Medical University (no. 29006) and conducted according to the ethical standards of the Declaration of Helsinki. Informed consent was obtained from the legal guardians of all patients included in the study.

### Data availability.

The DNA sequences generated in this study have been submitted to the GenBank database under accession numbers LC712443 to LC712625 (HRSV-A) and LC712626 to LC712733 (HRSV-B). The data sets generated and analyzed in this study are available from the corresponding author upon request.
