# Supplementary material for: Molecular Diversity of Human Respiratory Syncytial Virus before and during the COVID-19 Pandemic in Two Neighboring Japanese Cities
Source: Microbiol Spectr. 2023 Jul 6;11(4):e02606-22. doi: 10.1128/spectrum.02606-22 (PMC10433803; doi:10.1128/spectrum.02606-22)
Supplement: Supplemental file 1 — Supplemental material. Download spectrum.02606-22-s0001.pdf, PDF file, 1.1 MB [file spectrum.02606-22-s0001.pdf]

**Supplemental Material**

## FIGURES

**Fig. S1.** HRSV-A phylogenetic tree constructed from the sequences of the second HVR of *G* using the maximum likelihood method with the best model (GTR + G).

A phylogenetic tree was constructed using the maximum likelihood method with 1000 iterations for the bootstrap using MEGA version 7.0.26. Only bootstrap values greater than 70% are shown. The evolutionary distances were computed using the p-distance method and the scale bar represents the number of nucleotide substitutions per site. Strains detected at Hospital O are shown in red and those detected at Hospital H are shown in blue. The strains circulating in different countries are shown in purple. The representative strains of each genotype are shown in green.

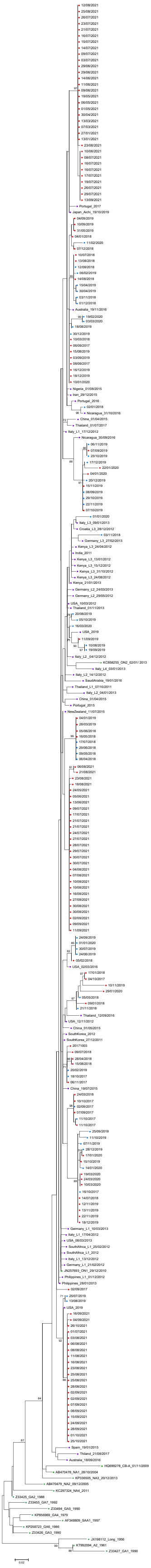
12/08/2021
25/08/2021
26/07/2021
23/07/2021
21/07/2021
16/07/2021
15/07/2021
14/07/2021
09/07/2021
03/07/2021
29/06/2021
29/06/2021
14/06/2021
11/06/2021
97
09/06/2021
19/05/2021
06/05/2021
01/05/2021
30/04/2021
13/03/2021
07/03/2021
27/01/2021
13/01/2021
23/06/2021
10/06/2021
08/07/2021
16/07/2021
16/07/2021
17/07/2021
19/07/2021
26/07/2021
29/07/2021
13/09/2021
Portugal_2017
Japan_Aichi_19/10/2019
04/09/2019
10/09/2019
31/05/2019
04/01/2018
11/02/2020
07/12/2018
10/07/2018
13/08/2018
12/09/2018
06/02/2019
85
14/08/2018
15/04/2019
30/04/2019
03/11/2018
01/12/2018
Australia_19/11/2016
99
19/02/2020
03/03/2020
18/08/2019
30/12/2019
10/03/2018
06/09/2017
15/08/2019
03/09/2019
08/09/2017
16/12/2019
18/12/2019
10/01/2020
Nigeria_01/08/2015
Iran_29/12/2015
Portugal_2016
02/01/2018
96
Nicaragua_31/10/2016
72
China_01/04/2015
Thailand_01/07/2017
Italy_L1_17/12/2012
Nicaragua_30/09/2016
86
06/11/2019
07/09/2019
23/10/2019
88
17/12/2019
22/01/2020
04/01/2020
20/12/2019
91
15/11/2019
08/09/2019
29/10/2019
22/11/2019
07/10/2019
01/01/2020
Italy_L3_09/01/2013
Croatia_L3_28/12/2012
03/11/2018
Germany_L3_27/02/2013
Kenya_L3_24/04/2012
India_2011
Kenya_L3_13/01/2012
Kenya_L3_15/12/2012
Kenya_L3_31/10/2012
Kenya_L3_24/08/2012
Kenya_21/01/2013
Germany_L2_24/03/2013
Germany_L2_29/05/2012
USA_10/03/2012
Thailand_01/11/2013
76
20/08/2019
05/10/2019
16/03/2020
USA_2019
71
11/09/2019
90
10/08/2019
97
19/09/2019
Italy_L2 _04/12/2012
KC858255_ON2_02/01/ 2013
Italy_L4_03/01/2013
Italy_L2_14/12/2012
SaudiArabia_19/01/2016
Thailand_L1_07/10/2011
Italy_L2_04/01/2013
China_01/04/2015
Portugal_2015
NewZealand_11/07/2015
04/01/2019
28/03/2019
05/06/2018
99
16/05/2018
17/07/2018
29/06/2018
09/05/2018
06/04/2018
81
06/08/2021
21/08/2021
23/08/2021
18/08/2021
24/05/2021
05/06/2021
13/06/2021
09/07/2021
17/07/2021
21/07/2021
21/07/2021
24/07/2021
27/07/2021
28/07/2021
29/07/2021
30/07/2021
30/07/2021
04/08/2021
07/08/2021
10/08/2021
10/08/2021
16/08/2021
27/08/2021
30/08/2021
30/08/2021
02/09/2021
09/09/2021
11/09/2021
24/09/2019
85
01/01/2020
82
30/07/2019
24/06/2019
05/02/2018
USA_02/03/2016
97
17/01/2018
04/10/2017
10/11/2019
29/01/2020
87
05/05/2018
09/01/2018
21/11/2018
Thailand_12/09/2016
USA_12/11/2012
China_01/05/2015
SouthKorea_2012
SouthKorea_27/12/2011
20171003
09/07/2018
86
28/04/2018
15/08/2018
71
20/02/2019
18/10/2017
06/11/2017
China_19/07/2015
24/03/2018
10/10/2017
98
02/09/2017
07/09/2017
11/10/2017
11/10/2017
25/09/2019
11/10/2019
07/11/2019
70
28/12/2019
17/01/2020
15/10/2019
14/01/2020
19/03/2020
88
24/03/2020
10/03/2020
16/10/2017
14/07/2018
12/11/2019
13/11/2019
22/11/2019
18/12/2019
Germany_L1_10/03/2013
Italy_L1_17/04/2012
USA_08/03/2013
SouthAfrica_L1_25/02/2012
SouthAfrica_L1_2012
Italy_L1_13/12/2012
Germany_L1_21/02/2012
JN257693_ON1_29/12/2010
Philippines_L1_01/12/2012
Philippines_28/01/2013
02/09/2017
71
25/07/2019
13/08/2019
USA_2019
16/09/2021
04/09/2021
26/10/2021
01/07/2021
03/08/2021
92
06/08/2021
08/08/2021
11/08/2021
16/08/2021
23/08/2021
25/08/2021
86
25/08/2021
28/08/2021
02/09/2021
84
02/09/2021
07/09/2021
08/09/2021
15/09/2021
24/09/2021
28/09/2021
01/10/2021
25/10/2021
87
Spain_19/01/2015
Thiland_21/08/2017
Australia_18/09/2016
HQ699278_CB-A_01/11/2009
AB470478_NA1_28/10/2004
KP336505_NA3_20/12/2013
AB470479_NA2_09/12/2005
KC297324_NA4_2011
Z33425_GA2_1988
Z33455_GA7_1992
Z33494_GA5_1990
KP856969_GA4_1979
AF348809_SAA1_1997
KP258723_GA6_1986
Z33426_GA3_1990
JX198112_Long_1956
99
KT992094_A2_1961
88
Z33427_GA1_1990
0.02

**Fig. S2.** Details of the time-scaled maximum clade credibility tree using the best model (GTR) of the second HVR of *G* from HRSV-A constructed using the Bayesian Markov chain Monte Carlo (MCMC) method under the exponential molecular clock. Only posterior probabilities greater than 0.8 are shown. Strains detected at Hospital O are shown in red and those detected at Hospital H are shown in blue. The strains circulating in different countries are shown in purple. Representative strains of each genotype are shown in green.

# HRSV-A

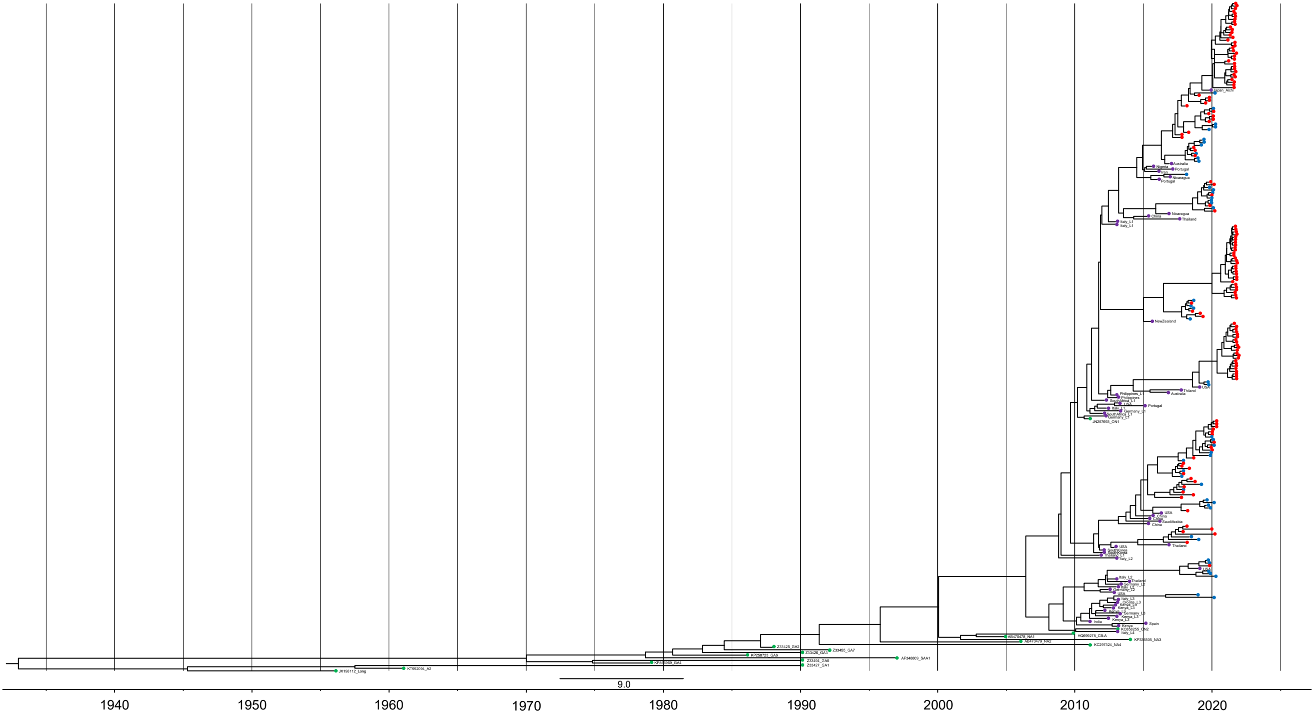

Japan_Aichi
Australia
Nigeria
Portugal
Iran
Nicaragua
Portugal
Nicaragua
China
Thailand
Italy_L1
Italy_L1
NewZealand
Thailand
USA
Australia
Philippines_L1
Philippines
SouthAfrica_L1
USA
Portugal
Italy_L1
Germany_L1
SouthAfrica_L1
Germany_L1
JN257693_ON1
USA
China
China
SaudiArabia
China
Thailand
USA
SouthKorea
SouthKorea
Thailand_L1
Italy_L2
USA
Italy_L2
Thailand
Germany_L2
Italy_L2
Germany_L2
USA
Italy_L3
Croatia_L3
Kenya_L3
Kenya_L3
Kenya_L3
Germany_L3
Kenya_L3
India
Kenya_L3
Spain
Kenya
KC858255_ON2
Italy_L4
HQ699278_CB-A
AB470478_NA1
AB470479_NA2
KP336505_NA3
KC297324_NA4
Z33425_GA2
Z33455_GA7
Z33426_GA3
KP258723_GA6
AF348809_SAA1
KP856969_GA4
Z33494_GA5
Z33427_GA1
KT992094_A2
JX198112_Long
9.0
1940
1950
1960
1970
1980
1990
2000
2010
2020

**Fig. S3.** HRSV-B phylogenetic tree constructed from sequences of the second HVR of *G* using the maximum likelihood method with the best model (GTR + G).

The phylogenetic tree was constructed using the maximum likelihood method with 1000 iteration for the bootstrap using MEGA version 7.0.26. Only bootstrap values greater than 70% are shown. Evolutionary distances were computed using the p-distance method and the scale bar represents the number of nucleotide substitutions per site. Strains detected at Hospital O are shown in red and those detected at Hospital H are shown in blue. The strains circulating in different countries are shown in purple. Representative strains of each genotype are shown in green.

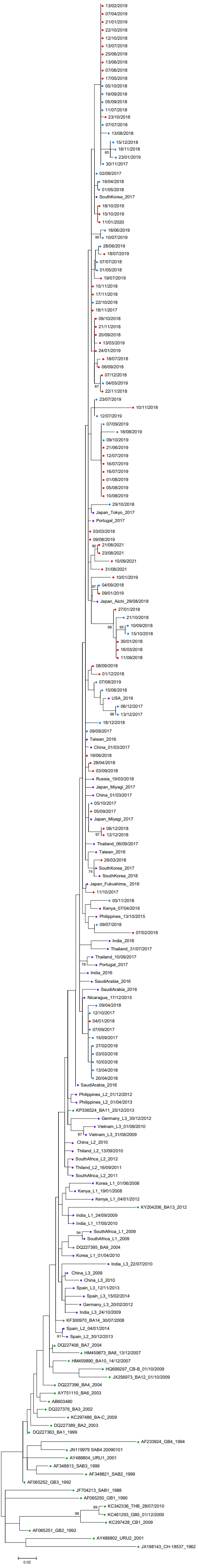
13/02/2019
07/04/2019
21/01/2019
22/10/2018
12/10/2018
13/07/2018
25/06/2018
13/06/2018
07/06/2018
17/05/2018
05/10/2018
19/09/2018
05/09/2018
11/07/2018
23/10/2018
07/07/2018
13/08/2018
15/12/2018
16/11/2018
85
23/01/2019
30/11/2017
02/09/2017
16/04/2018
01/05/2018
SouthKorea_2017
18/10/2019
15/10/2019
11/01/2020
18/06/2019
90
10/07/2019
28/06/2019
18/07/2019
07/07/2018
01/05/2018
19/07/2019
15/11/2018
17/11/2018
22/10/2018
18/11/2017
09/10/2018
21/11/2018
20/09/2018
13/03/2019
24/01/2019
18/07/2018
06/09/2018
07/12/2018
04/05/2019
87
22/11/2018
23/07/2019
10/11/2018
12/07/2019
07/09/2019
18/08/2019
09/10/2019
21/06/2019
12/07/2019
16/07/2019
16/07/2019
01/08/2019
05/08/2019
10/08/2019
29/10/2018
Japan_Tokyo_2017
Portugal_2017
03/03/2018
09/08/2019
90
21/08/2021
23/08/2021
10/09/2021
31/08/2021
10/01/2019
87
04/09/2018
09/01/2019
Japan_Aichi_29/08/2018
27/01/2018
21/10/2018
95
10/09/2018
98
15/10/2018
30/01/2018
16/03/2018
11/09/2018
08/09/2018
01/12/2018
07/08/2019
15/08/2018
USA_2019
06/12/2017
98
13/12/2017
18/12/2018
09/09/2017
Taiwan_2016
China_01/03/2017
19/06/2018
28/04/2018
03/09/2018
Russia_19/03/2018
Japan_Miyagi_2017
China_01/03/2017
05/10/2017
05/09/2017
Japan_Miyagi_2017
06/12/2018
97
12/12/2018
Thailand_06/09/2017
Taiwan_2016
74
26/03/2018
SouthKorea_2017
SouthKorea_2018
Japan_Fukushima_ 2016
11/10/2017
05/11/2018
Kenya_07/04/2016
Philippines_13/10/2015
09/07/2018
07/02/2018
India_2016
Thailand_31/07/2017
Thailand_10/09/2017
76
Portugal_2017
India_2016
SaudiArabia_2016
SaudiArabia_2016
Nicaragua_17/12/2015
09/04/2018
12/10/2017
04/01/2018
07/09/2017
15/09/2017
27/02/2018
03/03/2018
10/03/2018
13/04/2018
20/04/2018
SaudiArabia_2016
Philippines_L2_01/12/2012
Philippines_L2_01/04/2013
KP336524_BA11_20/12/2013
Germany_L3_30/12/2012
Vietnam_L3_01/09/2010
87
Vietnam_L3_31/08/2009
China_L2_2010
Thiland_L2_13/09/2010
SouthAfrica_L2_2012
Thailand_L2_16/09/2011
SouthAfrica_L2_2011
Korea_L1_01/06/2008
Kenya_L1_19/01/2008
Kenya_L1_04/01/2012
KY204206_BA13_2012
India_L1_24/09/2009
India_L1_17/05/2010
94
SouthAfrica_L1_2009
SouthAfrica_L1_2009
DQ227395_BA9_2004
Korea_L1_01/04/2010
India_L3_22/07/2010
China_L3_2009
China_L3_2010
Spain_L3_12/11/2013
Spain_L3_15/02/2014
Germany_L3_20/02/2012
India_L3_24/10/2009
KF300970_BA14_30/07/2008
Spain_L2_04/01/2014
91
Spain_L2_30/12/2013
DQ227406_BA7_2004
HM459873_BA8_13/12/2007
HM459890_BA10_14/12/2007
HQ699297_CB-B_01/10/2009
JX256973_BA12_01/10/2009
DQ227396_BA4_2004
AY751110_BA6_2003
AB603480
DQ227376_BA3_2002
KC297486_BA-C_2009
DQ227389_BA2_2003
DQ227363_BA1_1999
AF233924_GB4_1994
JN119979 SAB4 20090101
AY488804_URU1_2001
AF348813_SAB3_1999
AF348821_SAB2_1999
AF065252_GB3_1992
JF704213_SAB1_1988
AF065250_GB1_1990
99
KC342336_THB_28/07/2010
98
KC461293_GB5_01/12/2009
KC297428_CB1_2009
AF065251_GB2_1992
AY488802_URU2_2001
JX198143_CH-18537_1962
0.02

**Fig. S4.** Details of the time-scaled maximum clade credibility tree using the best model (GTR) of the second HVR of *G* from HRSV-B constructed using the Bayesian Markov chain Monte Carlo (MCMC) method under the exponential molecular clock. Only posterior probabilities greater than 0.8 are shown. Strains detected at Hospital O are shown in red and those detected at Hospital H are shown in blue. The strains circulating in different countries are shown in purple. Representative strains of each genotype are shown in green.

# HRSV-B

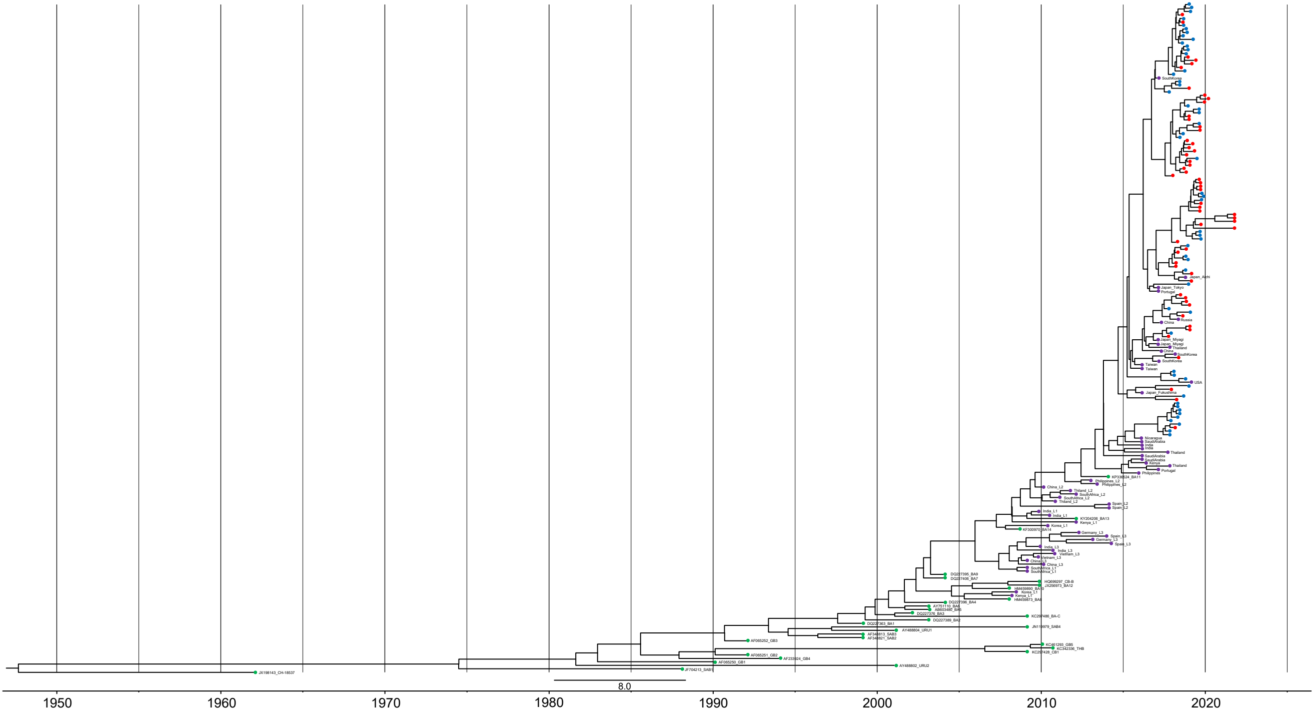

**TABLES**

**Table S1.** Evolutionary rates of the analyzed HRSV-A and HRSV-B strains.

| Subtype | Mean rate (95 % HPD) (substitution/site/year) |
|---|---|
| HRSV-A | $3.54 \times 10^{-3}$ ($2.74–4.37 \times 10^{-3}$) |
| HRSV-B | $6.02 \times 10^{-3}$ ($4.56–7.42 \times 10^{-3}$) |

**Table S2.** Ratio of the same array and pairwise distance per cluster of HRSV-A and HRSV-B detected at Hospital O in 2021.

| | HRSV-A | | | HRSV-B |
|---|---|---|---|---|
| Cluster | 1 | 3 | 4 | 2 |
| Ratio of same array | 23/33 and 9/33 | 24/28 and 2/28 | 20/22 | 2/4 |
| Pairwise distance | 0.006 | 0.012 | 0.006 | 0.02 |

Clinical strains of HRSV-A and HRSV-B in 2021 included several strains with exactly the same sequence in each cluster, and the pairwise distance was also low.

**Table S3.** Pairwise distance of HRSV-A and HRSV-B clusters detected at Hospitals O and H during 2017–21.

| | HRSV-A | | | | | | | |
|---|---|---|---|---|---|---|---|---|
| Cluster | 1 | 2 | 3 | 4 | 5 | 6 | 7 | 8 |
| Pairwise distance | 0.042 | 0.027 | 0.027 | 0.012 | 0.054 | 0.051 | 0.030 | 0.042 |

| | HRSV-B | | | | | |
|---|---|---|---|---|---|---|
| Cluster | 1 | 2 | 3 | 4 | 5 | 6 |
| Pairwise distance | 0.053 | 0.066 | 0.033 | 0.015 | 0.059 | 0.010 |

**Table S4.** Details of the sequences used for constructing phylogenetic trees using the maximum likelihood (ML) method, Bayesian Markov chain Monte Carlo (MCMC) method, and genome network analysis.

**Table S4.** Details of the sequences used for constructing phylogenetic trees using the maximum likelihood (ML) method, Bayesian Markov chain Monte Carlo (MCMC) method, and genome network analysis.

| Accession Number | Country/Year of isolation | Subgroup | Genotype | ML method | MCMC method | Network analysis |
|---|---|---|---|---|---|---|
| KT992094 | Australia/1961 | A | | ✓ | ✓ | |
| JX198112 | USA/1956 | A | | ✓ | ✓ | |
| Z33427 | Uruguay/1990 | A | GA1 | ✓ | ✓ | |
| Z33425 | Uruguay/1988 | A | GA2 | ✓ | ✓ | |
| Z33426 | Uruguay/1990 | A | GA3 | ✓ | ✓ | |
| KP856969 | USA/1979 | A | GA4 | ✓ | ✓ | |
| Z33494 | Uruguay/1990 | A | GA5 | ✓ | ✓ | |
| KP258723 | USA/1986 | A | GA6 | ✓ | ✓ | |
| Z33455 | Spain/1992 | A | GA7 | ✓ | ✓ | |
| AF348809 | South Africa/1997 | A | SSA1 | ✓ | ✓ | |
| AB470478 | Japan/2004 | A | NA1 | ✓ | ✓ | |
| AB470479 | Japan/2005 | A | NA2 | ✓ | ✓ | |
| KP336505 | China/2013 | A | NA3 | ✓ | ✓ | |
| KC297324 | China/2011 | A | NA4 | ✓ | ✓ | |
| HQ699278 | South Korea/2009 | A | CB-A | ✓ | ✓ | |
| JN257693 | Canada/2010 | A | ON1 | ✓ | ✓ | ✓ |
| KC858255 | Italy/2013 | A | ON2 | ✓ | ✓ | ✓ |
| JX198143 | USA/1962 | B | | ✓ | ✓ | |
| JF704213 | South Africa/1998 | B | SAB1 | ✓ | ✓ | |
| AF348821 | South Africa/1999 | B | SAB2 | ✓ | ✓ | |
| AF348813 | South Africa/1999 | B | SAB3 | ✓ | ✓ | |
| JN119979 | Cambodia/2009 | B | SAB4 | ✓ | ✓ | |
| AY488804 | Uruguay/2001 | B | URU1 | ✓ | ✓ | |

| | | | | | | |
|---|---|---|---|---|---|---|
| AY488802 | Uruguay/2001 | B | URU2 | ✓ | ✓ | |
| AF065250 | USA/1990 | B | GB1 | ✓ | ✓ | |
| AF065251 | USA/1992 | B | GB2 | ✓ | ✓ | |
| AF065252 | USA/1992 | B | GB3 | ✓ | ✓ | |
| AF233924 | USA/1994 | B | GB4 | ✓ | ✓ | |
| KC461293 | China/2009 | B | GB5 | ✓ | ✓ | |
| DQ227363 | Argentina/1999 | B | BA1 | ✓ | ✓ | |
| DQ227389 | Argentina/2003 | B | BA2 | ✓ | ✓ | |
| DQ227376 | Argentina/2002 | B | BA3 | ✓ | ✓ | |
| DQ227396 | Argentina/2004 | B | BA4 | ✓ | ✓ | |
| AB603480 | Japan/2003 | B | BA5 | ✓ | ✓ | |
| AY751110 | Belgium/2003 | B | BA6 | ✓ | ✓ | |
| DQ227406 | Argentina/2004 | B | BA7 | ✓ | ✓ | |
| HM459873 | Japan/2007 | B | BA8 | ✓ | ✓ | |
| DQ227395 | Argentina/2004 | B | BA9 | ✓ | ✓ | ✓ |
| HM459890 | Japan/2007 | B | BA10 | ✓ | ✓ | |
| KP336524 | China/2013 | B | BA11 | ✓ | ✓ | |
| JX256973 | Malaysia/2009 | B | BA12 | ✓ | ✓ | |
| KY204206 | Pakistan/2012 | B | BA13 | ✓ | ✓ | |
| KF300970 | Panama/2008 | B | BA14 | ✓ | ✓ | |
| KC297486 | China/2009 | B | BA-C | ✓ | ✓ | |
| KC297428 | China/2009 | B | CB1 | ✓ | ✓ | |
| HQ699297 | South Korea/2009 | B | CB-B | ✓ | ✓ | |
| KC342336 | Thailand/2010 | B | THB | ✓ | ✓ | |
| MK634184 | South Korea/2012 | A | ON1 | ✓ | ✓ | ✓ |
| KY654518 | Philippines/2013 | A | ON1 | ✓ | ✓ | ✓ |
| JX627336 | South Korea/2011 | A | ON1 | ✓ | ✓ | ✓ |
| KU950651 | USA/2012 | A | ON1 | ✓ | ✓ | ✓ |
| KX765956 | New Zealand/2015 | A | ON1 | ✓ | ✓ | ✓ |
| LC530050 | Japan/2019 | A | ON1 | ✓ | ✓ | ✓ |
| MK749913 | Nicaragua/2016 | A | ON1 | ✓ | ✓ | ✓ |
| MN306031 | USA/2019 | A | ON1 | ✓ | ✓ | ✓ |
| MH181987 | Kenya/2013 | A | ON1 | ✓ | ✓ | ✓ |
| MK749886 | Nicaragua/2016 | A | ON1 | ✓ | ✓ | ✓ |

| | | | | | | |
|---|---|---|---|---|---|---|
| MN630103 | USA/2016 | A | ON1 | ✓ | ✓ | ✓ |
| MN306048 | USA/2019 | A | ON1 | ✓ | ✓ | ✓ |
| KU950492 | USA/2012 | A | ON1 | ✓ | ✓ | ✓ |
| MH447954 | Thailand/2013 | A | ON1 | ✓ | ✓ | ✓ |
| KJ672448 | USA/2013 | A | ON1 | ✓ | ✓ | ✓ |
| KC731482 | India/2011 | A | ON1 | ✓ | ✓ | ✓ |
| MK749881 | Nicaragua/2015 | B | BA9 | ✓ | ✓ | ✓ |
| MN306044 | USA/2019 | B | BA9 | ✓ | ✓ | ✓ |
| LC385002 | Philippines/2015 | B | BA9 | ✓ | ✓ | ✓ |
| LC495297 | Japan/2018 | B | BA9 | ✓ | ✓ | ✓ |
| MN365581 | Kenya/2016 | B | BA9 | ✓ | ✓ | ✓ |
| LC488177 | Japan/2016 | B | BA9 | ✓ | ✓ | ✓ |
| JX912358 | Germany/2012 | A | ON1 | ✓ | ✓ | ✓ |
| JX912364 | Germany/2012 | A | ON1 | ✓ | ✓ | ✓ |
| KJ710375 | Germany/2013 | A | ON1 | ✓ | ✓ | ✓ |
| KJ710380 | Germany/2013 | A | ON1 | ✓ | ✓ | ✓ |
| KJ710396 | Germany/2013 | A | ON1 | ✓ | ✓ | ✓ |
| JX988443 | Italy/2012 | A | GA2 | ✓ | ✓ | ✓ |
| KC858199 | Italy/2012 | A | GA2/ON1 | ✓ | ✓ | ✓ |
| KC858208 | Italy/2012 | A | GA2/ON1 | ✓ | ✓ | ✓ |
| KC858249 | Italy/2012 | A | GA2/ON1 | ✓ | ✓ | ✓ |
| KC858250 | Italy/2012 | A | GA2/ON1 | ✓ | ✓ | ✓ |
| KC858211 | Italy/2013 | A | GA2/ON1 | ✓ | ✓ | ✓ |
| KC858222 | Italy/2013 | A | GA2/ON1 | ✓ | ✓ | ✓ |
| KC858256 | Italy/2013 | A | ON2 | ✓ | ✓ | ✓ |
| AB846651 | Philippines/2012 | A | ON1 | ✓ | ✓ | ✓ |
| KC476744 | South Africa/2012 | A | ON1 | ✓ | ✓ | ✓ |
| JX885730 | South Africa/2012 | A | ON1 | ✓ | ✓ | ✓ |
| KC342434 | Thailand/2011 | A | ON1 | ✓ | ✓ | ✓ |
| KF587941 | Kenya/2012 | A | ON1 | ✓ | ✓ | ✓ |
| KF587952 | Kenya/2012 | A | ON1 | ✓ | ✓ | ✓ |
| KF587961 | Kenya/2012 | A | ON1 | ✓ | ✓ | ✓ |
| KF587967 | Kenya/2012 | A | ON1 | ✓ | ✓ | ✓ |
| KF587976 | Kenya/2012 | A | ON1 | ✓ | ✓ | ✓ |

| | | | | | | |
|---|---|---|---|---|---|---|
| KF057865 | Croatia/2012 | A | ON1 | √ | √ | √ |
| KU254628 | China/2015 | A | ON1 | √ | √ | √ |
| KU254631 | China/2015 | A | ON1 | √ | √ | √ |
| KU254636 | China/2015 | A | ON1 | √ | √ | √ |
| MH760610 | Australia/2016 | A | ON1 | √ | √ | √ |
| MN122463 | Portugal/2015 | A | ON1 | √ | √ | √ |
| MN122526 | Portugal/2016 | A | ON1 | √ | √ | √ |
| MN122553 | Portugal/2017 | A | ON1 | √ | √ | √ |
| MH174872 | Iran/2015 | A | ON1 | √ | √ | √ |
| KU736774 | Nigeria/2015 | A | ON1 | √ | √ | √ |
| MH447710 | Thailand/2017 | A | ON1 | √ | √ | √ |
| MH760638 | Australia/2016 | A | ON1 | √ | √ | √ |
| KT326803 | Spain/2015 | A | ON1 | √ | √ | √ |
| KX009687 | China/2015 | A | ON1 | √ | √ | √ |
| MH388034 | Saudi Arabia/2016 | A | ON1 | √ | √ | √ |
| MH447708 | Thailand/2016 | A | ON1 | √ | √ | √ |
| MH447714 | Thailand/2017 | A | ON1 | √ | √ | √ |
| KF246588 | India/2009 | B | BA9 | √ | √ | √ |
| KF246630 | India/2010 | B | BA9 | √ | √ | √ |
| HQ711811 | South Africa/2009 | B | BA9 | √ | √ | √ |
| KC476954 | South Africa/2009 | B | BA9 | √ | √ | √ |
| HQ699298 | Korea/2008 | B | BA9 | √ | √ | √ |
| HQ699290 | Korea/2010 | B | BA9 | √ | √ | √ |
| KF156570 | Kenya/2008 | B | BA9 | √ | √ | √ |
| KP862466 | Kenya/2012 | B | BA9 | √ | √ | √ |
| KM402711 | Spain/2013 | B | BA9 | √ | √ | √ |
| KM402675 | Spain/2014 | B | BA9 | √ | √ | √ |
| KM873461 | Philippines/2012 | B | BA9 | √ | √ | √ |
| KM873497 | Philippines/2013 | B | BA9 | √ | √ | √ |
| KC342339 | Thailand/2010 | B | BA9 | √ | √ | √ |
| KC342345 | Thailand/2011 | B | BA9 | √ | √ | √ |
| KC477035 | South Africa/2011 | B | BA9 | √ | √ | √ |
| KC477096 | South Africa/2012 | B | BA9 | √ | √ | √ |
| KC297457 | China/2010 | B | BA9 | √ | √ | √ |

| | | | | | | |
|---|---|---|---|---|---|---|
| KJ939921 | Vietnam/2009 | B | BA9 | ✓ | ✓ | ✓ |
| JX079979 | Vietnam/2010 | B | BA9 | ✓ | ✓ | ✓ |
| KC297441 | China/2009 | B | BA9 | ✓ | ✓ | ✓ |
| KC297447 | China/2010 | B | BA9 | ✓ | ✓ | ✓ |
| KJ690596 | India/2009 | B | BA9 | ✓ | ✓ | ✓ |
| KF246602 | India/2010 | B | BA9 | ✓ | ✓ | ✓ |
| KM402683 | Spain/2013 | B | BA9 | ✓ | ✓ | ✓ |
| KM402745 | Spain/2014 | B | BA9 | ✓ | ✓ | ✓ |
| KJ710407 | Germany/2012 | B | BA9 | ✓ | ✓ | ✓ |
| JX967575 | Germany/2012 | B | BA9 | ✓ | ✓ | ✓ |
| MH447754 | Thailand/2017 | B | BA9 | ✓ | ✓ | ✓ |
| MH447779 | Thailand/2017 | B | BA9 | ✓ | ✓ | ✓ |
| MH447786 | Thailand/2017 | B | BA9 | ✓ | ✓ | ✓ |
| MN122639 | Portugal/2017 | B | BA9 | ✓ | ✓ | ✓ |
| MN122686 | Portugal/2017 | B | BA9 | ✓ | ✓ | ✓ |
| MG962341 | India/2016 | B | BA9 | ✓ | ✓ | ✓ |
| MH476342 | India/2016 | B | BA9 | ✓ | ✓ | ✓ |
| MK947336 | South Korea/2017 | B | BA9 | ✓ | ✓ | ✓ |
| MK947337 | South Korea/2017 | B | BA9 | ✓ | ✓ | ✓ |
| MK947352 | South Korea/2018 | B | BA9 | ✓ | ✓ | ✓ |
| LC474554 | Japan/2017 | B | BA9 | ✓ | ✓ | ✓ |
| LC474555 | Japan/2017 | B | BA9 | ✓ | ✓ | ✓ |
| MN163125 | China/2017 | B | BA9 | ✓ | ✓ | ✓ |
| MN163126 | China/2017 | B | BA9 | ✓ | ✓ | ✓ |
| MK481079 | Russia/2018 | B | BA9 | ✓ | ✓ | ✓ |
| LC324679 | Japan/2017 | B | BA9 | ✓ | ✓ | ✓ |
| MK361086 | Taiwan/2016 | B | BA9 | ✓ | ✓ | ✓ |
| MK361094 | Taiwan/2016 | B | BA9 | ✓ | ✓ | ✓ |
| KY711305 | Saudi Arabia/2016 | B | BA9 | ✓ | ✓ | ✓ |
| KY711306 | Saudi Arabia/2016 | B | BA9 | ✓ | ✓ | ✓ |
| KY711307 | Saudi Arabia/2016 | B | BA9 | ✓ | ✓ | ✓ |

88
